# Integrating metabolomic data with machine learning approach for discovery of Q-markers from Jinqi Jiangtang preparation against type 2 diabetes

**DOI:** 10.1186/s13020-021-00438-x

**Published:** 2021-03-19

**Authors:** Lele Yang, Yan Xue, Jinchao Wei, Qi Dai, Peng Li

**Affiliations:** 1 State Key Laboratory of Quality Research in Chinese Medicine, Institute of Chinese Medical Sciences, University of Macau, Macau, China; 2 Chengdu Institute for Food and Drug Control, Chengdu, China

**Keywords:** Jinqi Jiangtang, Backpropagation artificial neural network, Machine learning, Q-markers, Mass spectrometry, Metabolomics

## Abstract

**Background:**

Jinqi Jiangtang (JQJT) has been widely used in clinical practice to prevent and treat type 2 diabetes. However, little research has been done to identify and classify its quality markers (Q-markers) associated with anti-diabetes bioactivity. In this study, a strategy combining mass spectrometry-based untargeted metabolomics with backpropagation artificial neural network (BP-ANN)-based machine learning approach was proposed to screen Q-markers from JQJT preparation.

**Methods:**

This strategy mainly involved chemical profiling of herbal medicines, statistic processing of metabolomic datasets, detection of different anti-diabetes activities and establishment of BP-ANN model. The chemical features of seventy-eight batches of JQJT extracts were first profiled by using the untargeted UPLC-LTQ-Orbitrap metabolomic approach. The chemical features obtained which were associated with different anti-diabetes activities based on three modes of action were normalized, ranked, and then pre-selected by using ReliefF feature selection. BP-ANN model was then established and optimized to screen Q-markers based on mean impact value (MIV).

**Results:**

Optimized BP-ANN architecture was established with high accuracy of R > 0.9983 and relative low error of MSE < 0.0014, which showed better performance than that of partial least square (PLS) model (R^2^ < 0.5). Meanwhile, the BP-ANN model was subsequently applied to further screen potential bioactive components from the pre-selected chemical features by calculating their MIVs. With this machine learning model, 10 potential Q-markers with bioactivity were discovered from JQJT. The tested anti-diabetes bioactivities of 78 batches of JQJT could be accurately predicted.

**Conclusions:**

This proposed artificial intelligence approach is desirable for quick and easy identification of Q-markers with bioactivity from JQJT preparation.

**Supplementary Information:**

The online version contains supplementary material available at 10.1186/s13020-021-00438-x.

## Background

Pharmacologically active natural products, mainly derived from herbal medicines and dietary supplements, have been an abundant and continued resource for discovery of new drug leads [1]. However, there still remains much concerns about the quality control, underlying action mechanisms, side effects and potential drug-herb interactions of the complex phytochemicals of herbal medicines [2–4]. Therefore, the identification of chemical markers for quality control, particularly those contributing most to their therapeutic efficacy, is the key to understand the scientific basis for the therapeutic application of herbal medicines [5].

Many efforts have been devoted to the development of strategy for the quality control of herbal medicines [6–8]. The concept of quality marker (Q-marker), which refers to chemical markers reflecting therapeutic effects of herbal medicines, has gained great attention recently as an effective solution to quality control [6]. A LC–MS-based metabolomics method, namely chinmedomics, has been employed to uncover Q-markers from various herbal medicines [7]. An integrated strategy has been established to identify Q-markers from Schisandra chinensis (Turcz.) Baill based on target metabolomics using HPLC–MS/MS method [9]. However, due to the high complexity of the MS datasets obtained, researchers commonly focus on the analysis of a restricted number of identified compounds to screen Q-markers of interest. Therefore, various machine learning approaches with their powerful data processing and prediction capability have been adopted to screen the bioactive compounds and predict the different signatures of food and herbal samples [10–12]. Among them, artificial neural network (ANN) models outperform the others in the modeling of complex and non-linear relationships [13–15]. The well-trained ANN models have been employed to identify complex patterns from datasets, make real-time predictions and offer adaptive solutions in multiple fields, such as natural products, nanotechnology, and bioresource technology [14, 15].

**Fig. 1:**
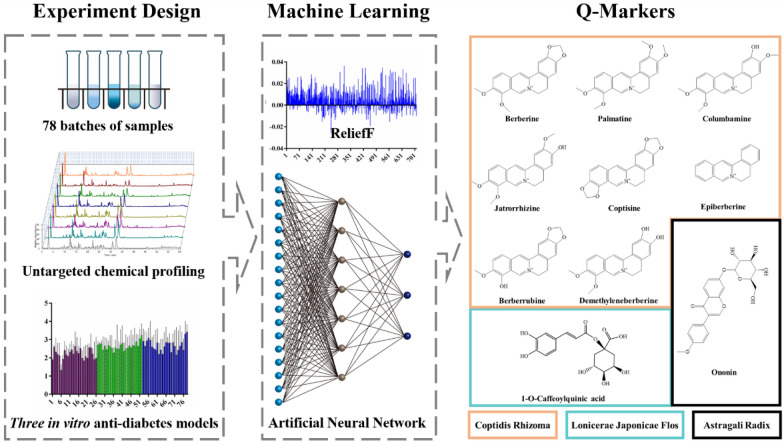
The proposed strategy for the discovery of bioactive compounds from Jinqi Jiangtang (JQJT) through mass spectrometry and backpropagation artificial neural network (BP-ANN)-based machine learning approach

Jinqi Jiangtang (JQJT), one of the anti-diabetic patent herbal formulas, is optimized from classic "Qianjin Huanglian pill". It consists of three herbal medicines Coptidis Rhizoma, Astragali Radix, and Lonicerae Japonicae Flos, which has been widely used in clinical practice to prevent and treat type 2 diabetes in China for decades [16]. Chemical profiles of JQJT result in discovery of several kinds of compounds, including alkaloids, flavonoids, phenolic acids and triterpene saponins etc. Many efforts have been made to identify the bioactive compounds and clarify the anti-diabetes mechanism, so as to establish a comprehensive understanding of its therapeutic effects [17–19]. However, limited information about its Q-markers associated with anti-diabetes bioactivity has been gathered from these research thus far. With consideration of this situation, we proposed a data-driven approach combining mass spectrometry-based untargeted metabolomics with backpropagation artificial neural network (BP-ANN)-based machine learning approach to screening Q-markers from JQJT. The schematic flow of this novel strategy is illustrated in Fig. 1. The proposed data-driven approach is expected to be proven rapid and accurate in the discovery of potential Q-markers from herbal medicines at one time.

## Methods

### Reagents and materials

Acetonitrile (HPLC grade) and ethanol (HPLC grade) were purchased from Merck (Darmstadt, Germany). 2-NBDG probe was purchased from Selleck Chemicals (Houston, USA). Formic acid (MS grade) was from Sigma-Aldrich (St. Louis, MO, USA). The reference compounds of coptisine (≥ 98.0%), berberine (≥ 98.0%), berberubine (≥ 98.0%), epiberberine (≥ 98.0%), chlorogenic acid (≥ 98.0%), palmatine (≥ 98.0%), jatrorrhizine (≥ 98.0%), demethyleneberberine (≥ 98.0%), 5-*O*-caffeoylquinic acid (≥ 98.0%), and 3, 5-*O*-dicaffeoylquinic acid (≥ 98.0%) were obtained from Chengmust Biological Technology Co., Ltd. (Sichuan, China). Ultra-pure water was produced by a Milli-Q water purification system (Milford, MA, USA).

### Preparation of sample solutions

Different batches of three herbal samples were collected from Bozhou and Chengdu Hehuachi medicinal herbs market in China, including 6 batches of Coptidis Rhizoma (Voucher specimen number: 1706121001–1706121006), 5 batches of Astragali Radix (Voucher specimen number: 1706122001–1706122005), and 6 batches of Lonicerae Japonicae Flos (Voucher specimen number: 1706123001–1706123006). All the samples were authenticated by Prof. Peng Li and deposited at State Key Laboratory of Quality Research in Chinese Medicine, University of Macau (Macau, China). The samples were homogenized and sieved through a No. 40 mesh. Total of 26 JQJT samples were prepared by combining the powders of three herbal medicines at a ratio of 10.3:15.4:61.8 (Coptidis Rhizoma: Astragali Radix: Lonicerae Japonicae Flos). The JQJT samples (2050 mg) were extracted by ultra-sounded for 60 min with 10%, 50% or 90% ethanol (20 mL), respectively. After freeze-drying, the obtained dry extract was suspended in 10 mL water (equal to 205 mg /mL). Then, 78 batches of JQJT samples were produced. The extracts were centrifuged, and the supernatant was filtrated through a 0.22 µm filter (Millipore, USA). An aliquot of 10 μL resulted filtrate was subject to UPLC-LTQ-Orbitrap analysis.

### Untargeted metabolomic profiling

The chromatographic separation was performed on a C18 column (4.6 mm × 250 mm, 5 µm, Shiseido Co., Ltd., Tokyo, Japan) using Dionex UltiMate 3000 UPLC system (Thermo Scientific, SanJose, CA, USA). The column was maintained at 27 °C and eluted with mobile phase consisting of 0.1% formic acid water (A) and 0.1% formic acid acetonitrile (B) under the following gradient program: 8%-12% B (0–5 min), 12–18% B (5–10 min), 18–22% B (10–15 min), 22–25% B (15–20 min), 25–32% B (20–35 min), 32–65% B (35–50 min), 65–95% B (50–60 min) at a flow rate of 1.0 mL/min. The column was equilibrated for an additional 5 min at 8% B (1.0 mL/min) after a gradient run. And 25% of column effluent was introduced into the ESI source via a post-column flow splitter (Analytical Scientific Instruments, CA, USA).

Mass spectrometry was performed on an LTQ-Orbitrap system (Thermo Fisher Scientific, Bremen, Germany) equipped with a heated electrospray ionization (ESI) source operating in the positive ionization mode. The key operating parameters of MS were as follows: spray voltage of 3.2 kV, sheath gas of 20 (arbitrary units); capillary temperature of 350 °C; auxiliary gas of 10 (arbitrary units), sweep gas of 2 (arbitrary units), and capillary voltage of 25 V. Full scan mass spectra were acquired in the mass range of m/z 100 to 1500 with a resolving power of 30,000. Data-dependent MS/MS fragmentation was performed to acquire MS^2^ spectra in linear ion trap. Dynamic exclusion was used to avoid repeated MS/MS analysis with the exclusion time of 30 s. The collision-induced dissociation (CID) was used with normalized collision energy of 35%. The data acquiring and processing was performed using Thermo Xcalibur 2.1 (Thermo Fisher Scientific) workstation.

### Metabolomics data processing

Raw data (.raw format) acquired with Xcalibur workstation were converted to the mzXML data using the MSConvert software. XCMS online, an open-source deconvolution software, was employed to pretreat the obtained mzXML [20]. After peak extracting, filtering and alignment, the dataset containing the integrated peak intensity, m/z, and retention time was obtained. MetaboAnalyst 3.0 online algorithm, a web-based tool designed for data normalization (normalized by median), visualization and interpretation, was applied for further multivariate analysis of the peak table by using the statistical analysis module with default parameters [21].

PCA was then performed on these normalized data by using the statistical analysis module in Metaboanalyst as to discriminate the metabolomic differences among different herbal medicines and JQJT samples, respectively [22]. The normalized data were subsequently imported into the SIMCA-P 13.0 platform (Umetrics AB, Umeå, Sweden) for the partial least square (PLS) analysis to screen the chemical features with anti-diabetic activity. R^2^ value, namely the determination coefficient, was used to estimate the predictive ability of the established model.

### Bioactivity assay

Type 2 diabetes is a long-term metabolic disorder associated with elevated blood glucose levels, insulin resistance, and relative lack of insulin [23]. Glucose consumption, α-glucosidase inhibitory and uptake of 2-NBDG assays are widely used to screen anti-diabetes compounds from natural products [24]. In order to classify the types of the bioactivities of the effective components, the three in-vitro anti-diabetes models were employed.

#### Glucose consumption

Glucose consumption was determined by using a murine hepatocyte AML12 cell line purchased from American Type Culture Collection (ATCC, Rockville, MD, USA). AML12 cells were cultured in 96-well plates with 10^4^ cells/well and starved in 100 μL DMEM/F12 (17.5 mM glucose) for 6 h, then treated with 1 μL of 205 mg/mL JQJT solutions or 100 mM metformin. After 16 h, glucose concentrations in the culture supernatant were determined with glucose assay kit (Nanjing Jiancheng Bioengineering Institute, Nanjing, China) and glucose consumption were calculated as described previously [25].

#### α-Glucosidase inhibition

The inhibition effect of JQJT preparation against α-glucosidase enzyme was measured according to previous study [26]. α-Glucosidase and substrate (4-nitrophenyl α-d-glucopyranoside, p-NBDG) were purchased from Sigma-Aldrich. Briefly, α-glucosidase and p-NBDG were dissolved in potassium phosphate buffer (67 mM, pH 6.8). Reaction mixture containing 10 μL of sample solutions (1.3 mg/mL), 40 μL of α-glucosidase (0.25 U/mL) solution was pre-incubated at 37 °C for 15 min. Then, 135 μL of 4 mM p-NBDG was added into the mixture and incubated for 30 min at 37 °C. The enzymatic reaction was terminated by the addition of 75 μL of sodium carbonate solution (0.2 mol/L). The α-glucosidase inhibition activity of tested samples was evaluated by measuring the absorbance (Abs) at 405 nm. Acarbose (2000 µg/mL) was used as the positive control. The inhibitory potency against α-glucosidase was calculated as follows: Inhibition% = [(Abs control − Abs sample) / Abs control].

#### Glucose uptake assay

Cellular glucose uptake was determined using the fluorescent probe 2‐NBDG according to previous method [27]. Palmitate was dissolved in ethanol and mixed with fatty acid-free bovine serum albumin (BSA) stirred at 50 °C for 2 h [28]. L6 myotubes (ATCC) were seeded into 96-well cell culture plates (10^4^ cells/well in 100 μL culture medium) and pre-treated with 1 μL of 205 mg/mL test samples or metformin (100 mM) for 2 h, followed by incubation with 0.1 μL of 0.5 M palmitic acid-BSA conjugate solution for 16 h. Cells were starved with culture medium for 2 h, and then incubated with or without 100 μL of 100 μM 2-NBDG for 30 min. The intensity of fluorescence was measured after incubation with 0.1 μL of 100 μM insulin for 10 min.

### Chemometric analysis

#### Chemical features ranking and pre-selecting using ReliefF

The peak matrix extracted from the raw data was first processed with the ReliefF-based feature selection algorithm [29]. The weights of features were calculated and ranked using ReliefF algorithm. The top 18 ranked chemical features, which fit better with the bioactivities, were selected for subsequent BP-ANN modelling. ReliefF algorithm was performed within the MATLAB (R2016a, Mathworks, Natick, USA).

#### BP-ANN model

BP algorithm minimizes the error in predictions and produces satisfactory results by adjusting each weight of the networks, which was utilized in the establishment of ANN model [30]. In the current study, the inputs for the BP-ANN were the pre-selected peaks intensities, while the three bioactivities were set as output. As showed in Additional file 1: Fig. S1, BP-ANN consisted of 18 neurons as the input layer, 7 neurons as the hidden layer and 3 neurons as the output layer. In modeling, 70% of the total samples were used and treated as the training data set for building the model. To prevent overfitting, 15% of the samples were used as the validation data set for early stopping methodology. The 15% remained were used as the test data for testing the model and cross-validation. Based on the preliminary experiment, the dividerand function was chosen to randomly divide the sample data in this work. The mean square error (MSE) was employed to estimate the performance of BP-ANN model during the training process. ANN tool of MATLAB was used to build the BP-ANN model.

### Structural elucidation of selected compounds

The Xcalibur 2.1 was used to process the raw MS data. The compounds responsible for the bioactivity were screened and further identified. Mass Frontier 7.0 (Thermo Fisher Scientific, San Jose, CA, USA) was employed for the structural identification and the proposed of fragmentation patterns. The results were confirmed by relevant literature data or comparing their retention times with reference standards.

## Results

### Untargeted metabolomic profiling of different JQJT combinations

**Fig. 2:**
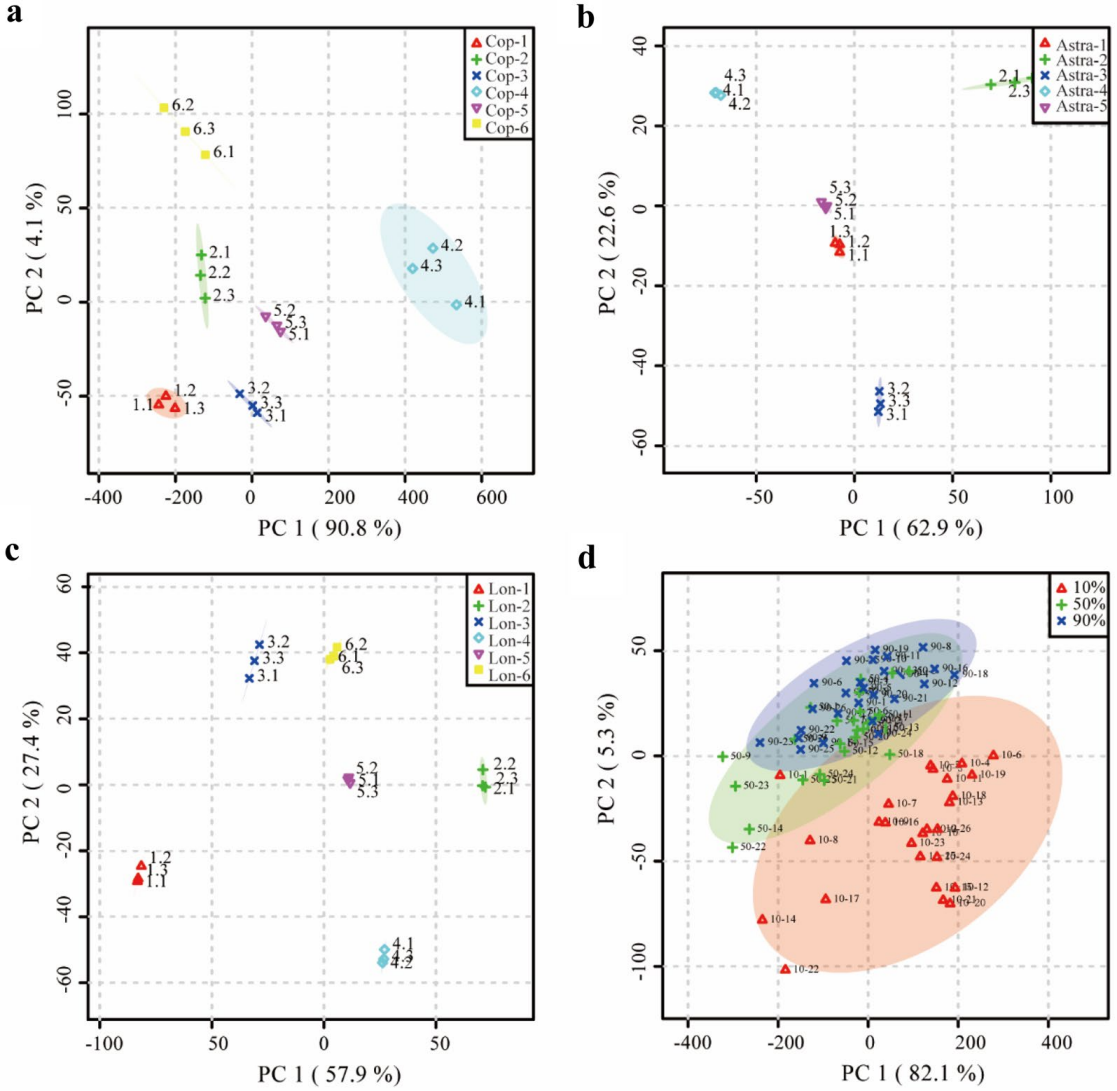
The PCA score plots of different samples. **a** Coptidis Rhizoma (Cop); **b** Astragali Radix (Astra); **c** Lonicerae Japonicae Flos (Lon); **d** Different batches of JQJT samples extracted with 10% (red), 50% (green), and 90% (blue) ethanol

Different batches of the three herbal medicines collected from diverse origins were profiled respectively by using the developed UPLC-LTQ-Orbitrap method. The typical total ion chromatograms of the three herbal medicines were depicted in Additional file 1: Fig. S2, and the differences of peak intensity were significant for discriminating them. As illustrated in PCA score plot (Fig. 2a–c), almost all herbal medicines were grouped into different clusters. Untargeted metabolomics were then carried out to profile the chemical composition of the total 78 different JQJT extracts, respectively. After deconvolution, a total of 1010 peaks were obtained. Considering that a large amount of missing values may result in low power for downstream analysis, we performed missing value estimation, data filtering and normalization via MetaboAnalyst using default parameters prior to the multivariate statistical analysis, with 710 ions retained for the following statistical analysis [31]. To uncover the similarities among samples and detect the outliers, further exploration of the processed data was made by using PCA. On the score plot of PCA (Fig. 2d), different JQJT were not clearly classified into three separated regions, implying the redundancy of 710 features.

### Bioactivities of different JQJT combinations

**Fig. 3:**
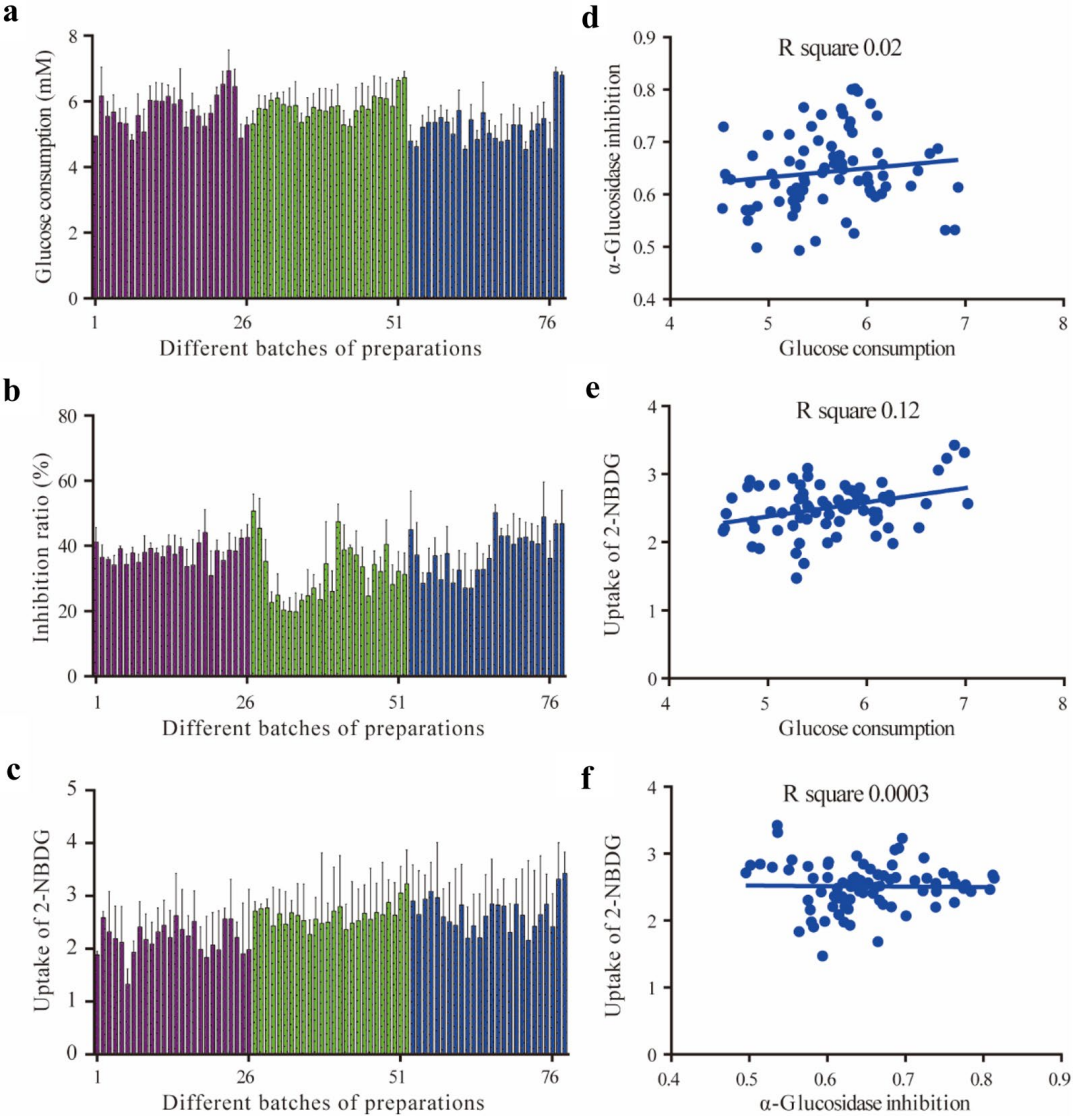
Anti-diabetes capacities of 78 batches of JQJT samples and their correlation. **a** Promotion of glucose consumption; **b** Inhibition of α-glucosidase; **c** Promotion of 2-NBDG uptake; **d** Correlation between **a** and **b**; **e** Correlation between **a** and **c**; **f** Correlation between **b** and **c**

Different signaling pathways associated with the anti-diabetes effect of JQJT have also been reported [17], such as the inhibitory activity against α-glucosidase, α-amylase, preventing insulin resistance, and promoting glucose uptake etc. Herein, three different anti-diabetes models, promotion of glucose consumption, inhibition of α-glucosidase, and enhancement of glucose uptake, were used to examine the bioactivities of JQJT samples. As shown in Fig. 3a–c, the values of three bioactivities ranged from 4.53 to 6.93 mM, 20% to 51%, and 1.47 to 3.43, respectively. Most tested samples showed average values, as is evident in the figure. In addition, no significant linear correlation with R^2^ ≤ 0.12 among the three assays were observed (Fig. 3d–f). These results indicated that the tested anti-diabetes activities were suitable to screen bioactive compounds associated with different anti-diabetes activities.

### Discovery of Q-markers based on machine learning techniques

#### Chemical features ranking and pre-selecting using ReliefF

**Fig. 4:**
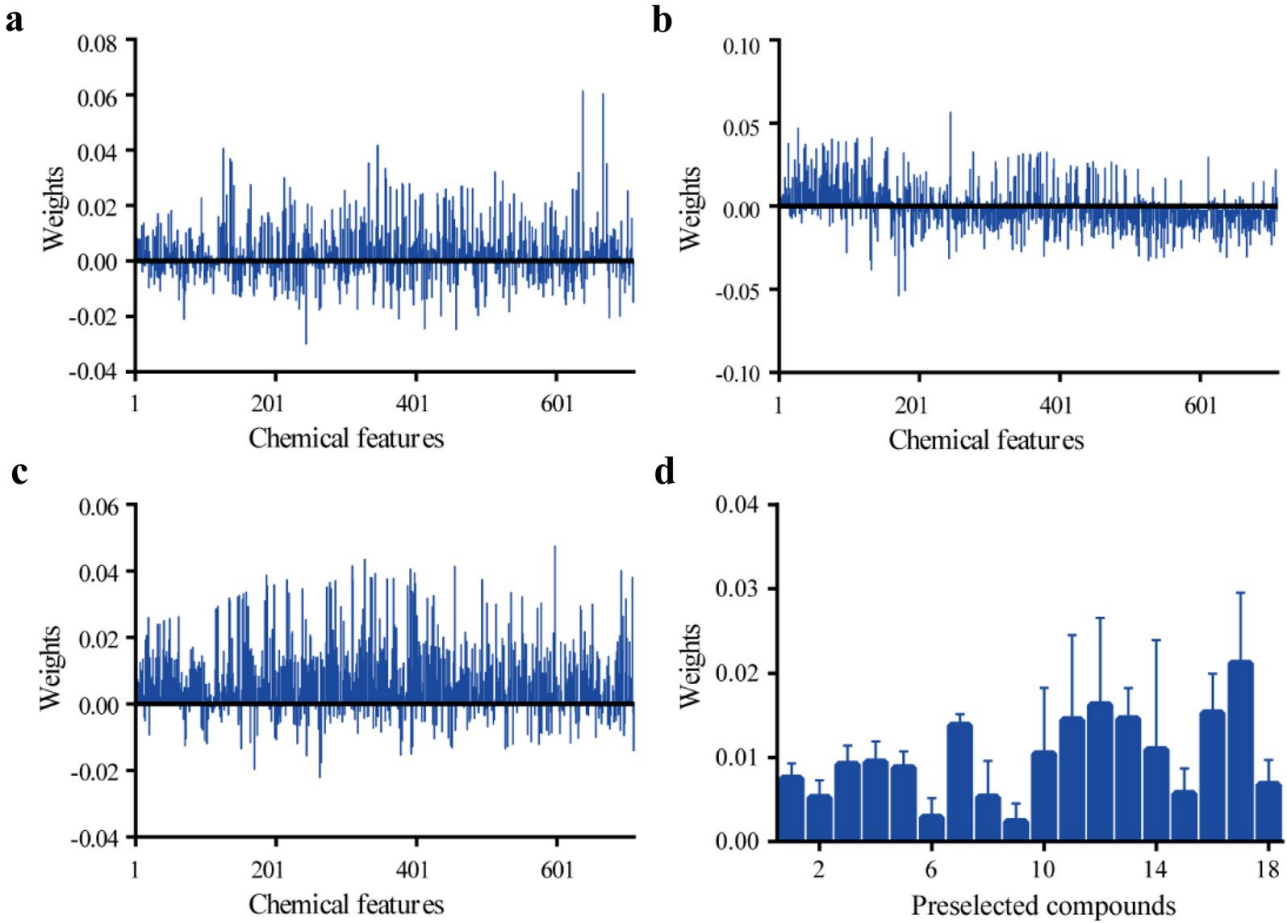
The weight scores of all chemical features calculated by ReliefF. **a** Glucose consumption; **b** α-Glucosidase inhibition; **c** Uptake of 2-NBDG; **d** pre-selected chemical features

We subsequently focused on the chemometric analysis for discovery of potential bioactive compounds. On one hand, processing of all the pre-processed data with high dimensionality would generate a rather complex statistical model with relatively low accuracy. Therefore, when all the processed datasets were firstly submitted to Simica-P for PLS analysis, the results only exhibited poor explanatory power for anti-diabetes activities, in which the established PLS model with R^2^ ≤ 0.2 and coefficient ≤ 0.004 (Additional file 1: Fig. S3). On the other hand, the introduction of extra parameters probably leads to overfitting of chemometric model. Thus, in the end, a ReliefF algorithm-based feature selection technique was employed to select a subset of representative chemical features in this work. The feature importance ranked by ReliefF algorithm was displayed in Fig. 4a–c, respectively. The chemical features with a higher weight value were closely correlated with the bioactivity. To identify potential bioactive compounds rapidly, we collected the structural information of metabolites in JQJT from databases like PubChem, METLIN and MassBank, etc. The top 18 identified chemical features with high weights (Fig. 4d) were pre-selected to build a BP-ANN model in this case.

#### Establishment of BP-ANN model

A total of 14 training algorithms were tested (Additional file 1: Table S1), the results revealed that the Bayesian regularization training algorithm worked as an efficient method to accurately simulate and predict the three different bioactivities. The hidden layer was assigned with increasing number of neurons, and then the neuron numbers were determined by comparing the resulted MSE. The neuron numbers in the hidden layer were analyzed between 1 and 10. As the results in Additional file 1: Table S2 show, the number of hidden layers was 1, and neuron numbers in the hidden layer were 7. Three transfer functions including purelin, logsig, and tansig were selected for the comparison of the performance of this model. The final applied training function was the tansig (Additional file 1: Table S2). And following the key selection of training algorithm, initial bias, initial weights, and mu decrease factor were further optimized by using response surface methodology (RSM) to construct a precise model (Additional file 1: Table S3).

#### Performance of the BP-ANN model

**Fig. 5:**
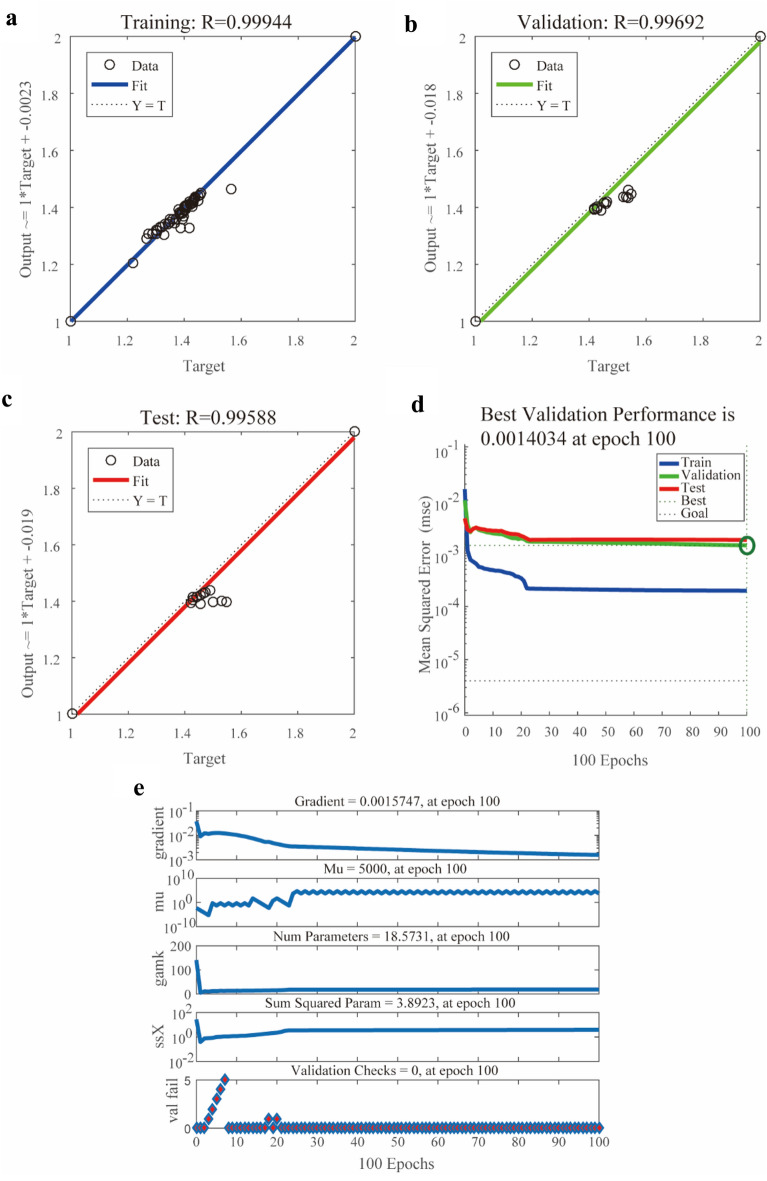
Performance of the established BP-ANN model in training (**a**), test (**b**) and validation (**c**) steps. The plot of MSE (**d**), magnitude of the gradient, and the number of validation checks (**e**) on the establishment of BP-ANN model

The final achieved BP-ANN model showed desirable performance in predicting and simulating bioactivities. The satisfactory performance demonstrated with the correlation plots was observed as shown in Fig. 5a–c, where R of the training, validation, and test data set reached 0.9994, 0.9969 and 0.9959, respectively. In addition, the training process of BP-ANN and the checking of network performance were evaluated by the value of MSE (Fig. 5d), the magnitude of the gradient and the number of validation checks (Fig. 5e). The value of R in this model was 0.9983, which suggested that the overall experiment values corresponded well with the predicted data. To further confirm the better performance of established BP-ANN model in predicting anti-diabetes activities and cross validate the potential bioactive compounds, the pre-selected bioactive compounds were used to construct abovementioned PLS model based on the same 78 samples. Results (Additional file 1: Fig. S4) indicated that the performance of BP-ANN model developed in this study was better than PLS model (R^2^ < 0.90). The mean impact value (MIV) of input variables in the networks were employed to evaluate the importance of different variables in the use of BP-ANN model [32]. The obtained MIVs (Additional file 1: Table S4) showed that 10 out of the 18 pre-selected compounds exhibited potential anti-diabetes activity.

### Identification of Q-markers based on the summed MIVs

**Fig. 6:**
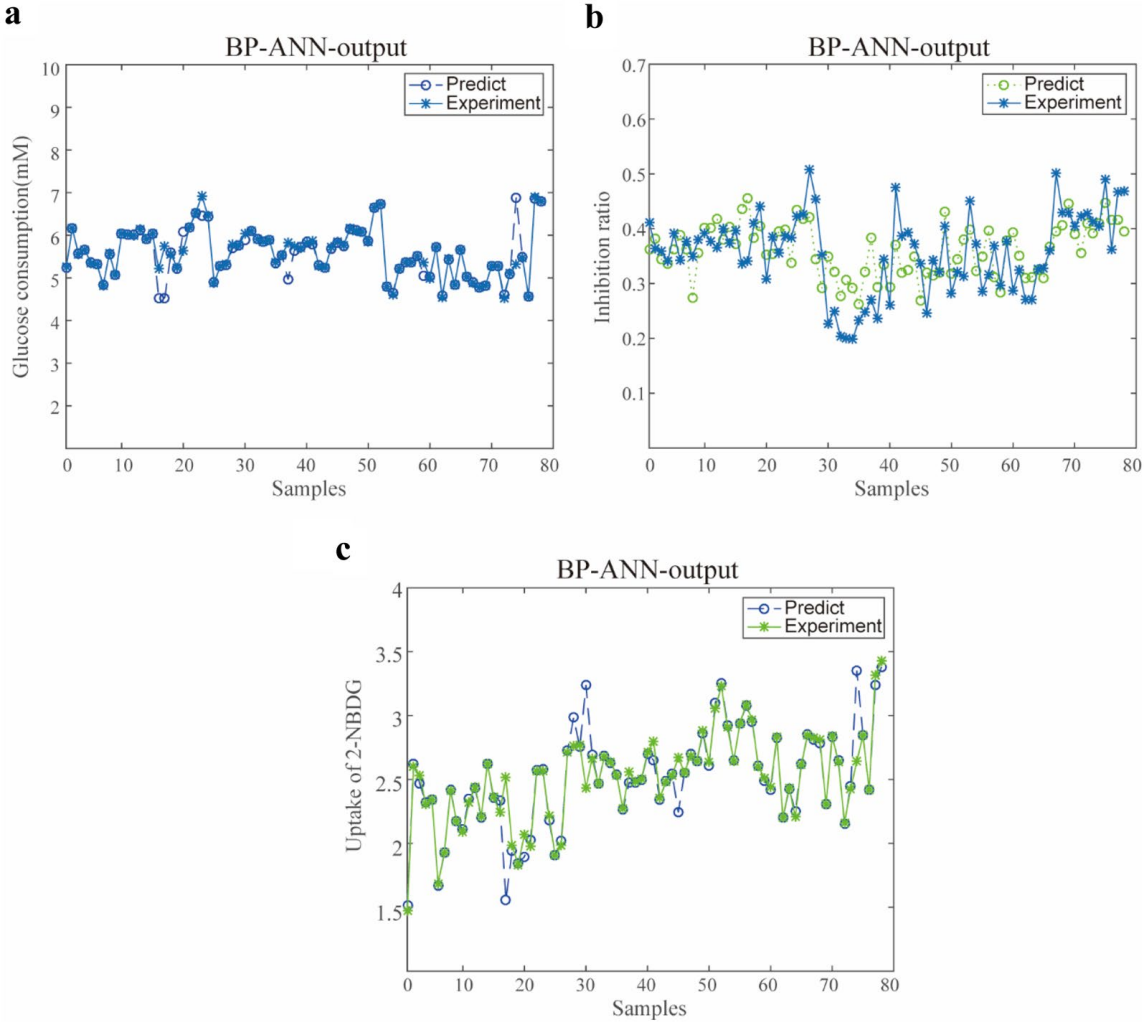
The visual agreements between experimental and predicted data sets (**a** Glucose consumption; **b** α-Glucosidase inhibition; **c** Uptake of 2-NBDG)

The potential Q-markers were finally screened based on the summed MIVs in the BP-ANN model. With this approach, a total of 10 compounds with summed MIV > 0, mainly consisting of 8 alkaloids, 1 phenolic acid, and 1 flavonoid were screened and further identified as displayed in Table 1. Further comparison analysis of the predicted values with the experimental values was performed to estimate the overall performance of the 10 screened compounds with the BP-ANN model. The anti-diabetes activity could be predicted accurately from the screened Q-markers, which produced a reliable prediction for the whole dataset as revealed by the plot in Fig. 6a–c. Based on the relative abundant of the screened compounds, a heatmap visualization used for unsupervised clustering was constructed. As displayed in Additional file 1: Fig. S5, the content distribution of the screened compounds showed no obvious regularity in the 78 batches of samples. Additionally, the content difference of components in various samples could explain the different levels of anti-diabetes activity.

**Table 1:** Screened potential Q-markers based on mean impact value

Selected compounds	Compound name	Formula	tR /min	Experimental m/z	Theoretical m/z	Common fragment ions
S1	Berberine	C20H18NO4	31.87	*336.1216*	*336.123*	*321.1041, 320.0832, 306.0733, 304.0941, 292.0941*
S2	Palmatine	C21H22NO4	30.34	*352.1514*	*352.1543*	*338.1273, 337.1271, 336.1124, 322.1271, 308.1417, 291.1622*
S3	Columbamine	C20H20NO4	24.85	*338.1353*	*338.1387*	*324.1187, 323.1189, 308.1561, 306.0932, 294.1225, 293.0972*
S4	Jatrorrhizine	C20H20NO4	25.64	*338.1353*	*338.1387*	*324.1187, 323.1189, 308.1208, 295.1436*
S5	Coptisine	C19H14NO4	26.47	*320.089*	*320.0917*	*318.0861, 293.1106, 292.0846, 290.0876, 264.1363*
S6	Epiberberine	C20H18NO4	25.31	*336.1216*	*336.123*	*321.1094, 320.0993, 308.1068, 292.0815*
S7	Berberubine	C19H16NO4	12.70	*322.1064*	*322.1064*	*307.0826*
S8	Ononin	C22H22O9	28.2	*431.1305*	*431.1337*	*269.1364*
S12	1-O-Caffeoylquinic acid	C16H18O9	16.21	*355.099*	*355.0945*	*267.0846, 163.0635*
S18	Demethyleneberberine	C19H18NO4	22.18	*324.1209*	*324.1231*	*307.0814, 294.0844, 279.0705, 266.0983*

## Discussions

Herbal medicines are characterized by multiple components co-existing in a prescription and taking effects via a multi-target additive, synergistic, and/or competitive mode [25]. JQJT has been widely used in clinical practice to prevent and treat type 2 diabetes through multi-targets integrated mechanism [17]. Although considerable efforts have been implemented in the characterization of complex chemical compositions and metabolites of JQJT, various strategies to rapidly and accurately mine Q-markers associated with bioactivity are in urgent need. In this work, 10 potential Q-markers for JQJT were screened based on machine learning analysis of the established BP-ANN model.

Different data pre-processing (feature selection) models have been considered and selected prior to machine learning analysis, such as principal components analysis (PCA), naive Bayes, ReliefF, random forest, and support vector machines. They could help to extract key chemical information directly from highly complex datasets [20, 33, 34]. Among them, ReliefF algorithm evaluates the weight of each input variable and then ranks the variables based on their power to predict the target variable. This algorithm, not considering each input variable individually, weights predictive strength in terms of their interactions with other variables [35]. In this study, ReliefF was then applied for preliminarily ranking and selecting the peaks associated with anti-diabetes activities. Sample size requirement to successfully train and utilize ANN depends greatly upon the number of the features [36]. Considering the relatively small sample size, 18 compounds were selected for subsequent modelling. After that, BP-ANN model was established and optimized to evaluate the correlations between the selected peak data and the bioactivities data. It showed excellent explanatory ability for complex interactions between input and target variable, which is consistent with previous studies [37]. Finally, MIV was calculated to further screen the potential Q-markers with bioactivities, and their structures were identified based on the corresponding mass spectra.

Using this machine learning model, 10 potential Q-markers with bioactivity were discovered from JQJT. According to the definition of Q-marker, the screened Q-markers were supposed to reflect the efficacy of JQJT. Indeed, most of the screened chemical markers represent the “king (Jun)” herb-Coptidis Rhizoma by using this developed method [38]. Eight compounds including berberine, palmatine, columbamine, jatrorrhizine, coptisine, epiberberine, berberrubine, and demethyleneberberine were the chemical markers from Coptidis Rhizoma. In consistence with the results reported in the previous studies, some of the identified compounds exhibited anti-diabetes activity through different mechanisms. With this result of the respective MIVs, berberine, palmatine, jatrorrhizine, epiberberine, and berberubine were assigned as the potential bioactive compounds in terms of promotion effect on glucose consumption [39]. Chlorogenic acid was screened as anti-diabetes compound, which has been proven to be responsible for inhibitory potential against α-glucosidase [18]. Besides, ten compounds including berberine, columbamine, coptisine, ononin, 5-*O*-caffeoylquinic acid, loganic acid, 5-*O*-caffeoylquinic acid, 3,5-*O*-dicaffeoylquinic acid, luteolin-7-O-β-D-glucopyranoside and demethyleneberberine were more responsible for the promotion of glucose uptake [40]. Notably, 5-*O*-caffeoylquinic acid, luteolin-7-*O*-β-D-glucopyranosidea and chlorogenic acid, the three compounds associated with ameliorate effects on α-glucosidase inhibition or glucose uptake were explored for the first time. However, this strategy linked the bioactivity of JQJT only to the high-abundant components in the formula rather than the binding constants, which may omit the low-abundant components with greater binding affinities.

## Conclusions

This study reports a strategy for screening Q-markers with potential anti-diabetes activities by combining high resolution mass spectrometry-based untargeted metabolomics and BP-ANN-based machine learning approach. With the use of this strategy, 10 components were selected and identified as potential Q-markers. The information generated from the current study might pave the way for the preparation and quality control of JQJT extracts in pharmaceutical industry and clinical practice. Further studies would focus on the isolation of the screened compounds, and further confirmation of their anti-diabetes activities and possible synergistic mechanisms.

## Supplementary Information

**Additional file 1.** Additional tables and figures.

## Data Availability

The datasets used during this study are available from the corresponding author upon reasonable request.

## Abbreviations

Q-marker: Quality marker
PCA: Principal components analysis
ANN: Artificial neural network
BP-ANN: Backpropagation artificial neural network
JQJT: Jinqi Jiangtang
MIV: Mean impact value
CID: Collision-induced dissociation
PLS: Partial least square
RSM: Response surface methodology
MSE: Mean square error
