# Supplementary material for: Integrating metabolomic data with machine learning approach for discovery of Q-markers from Jinqi Jiangtang preparation against type 2 diabetes
Source: Chin Med. 2021 Mar 19;16:30. doi: 10.1186/s13020-021-00438-x (PMC7980607; doi:10.1186/s13020-021-00438-x)
Supplement: Supplementary file 1 — Additional file 1. Additional tables and figures. [file 13020_2021_438_MOESM1_ESM.docx]

**Supplementary Information**

**Integrating metabolomic data with** **machine learning approach for discovery of Q-markers from Jinqi Jiangtang preparation against type 2 diabetes**

Lele Yang **^1,†^**, Yan Xue **^1,†^**, Jinchao Wei **^1^**, Qi Dai **^2^**, and Peng Li **^1,*^**

**^1^** State Key Laboratory of Quality Research in Chinese Medicine, Institute of Chinese Medical Sciences, University of Macau, Macau, China

**^2^** Chengdu Institute for Food and Drug Control, Chengdu, China

**^*^** Correspondence: pli1978@hotmail.com (P.L.)

**^†^** These authors contributed equally to this work.


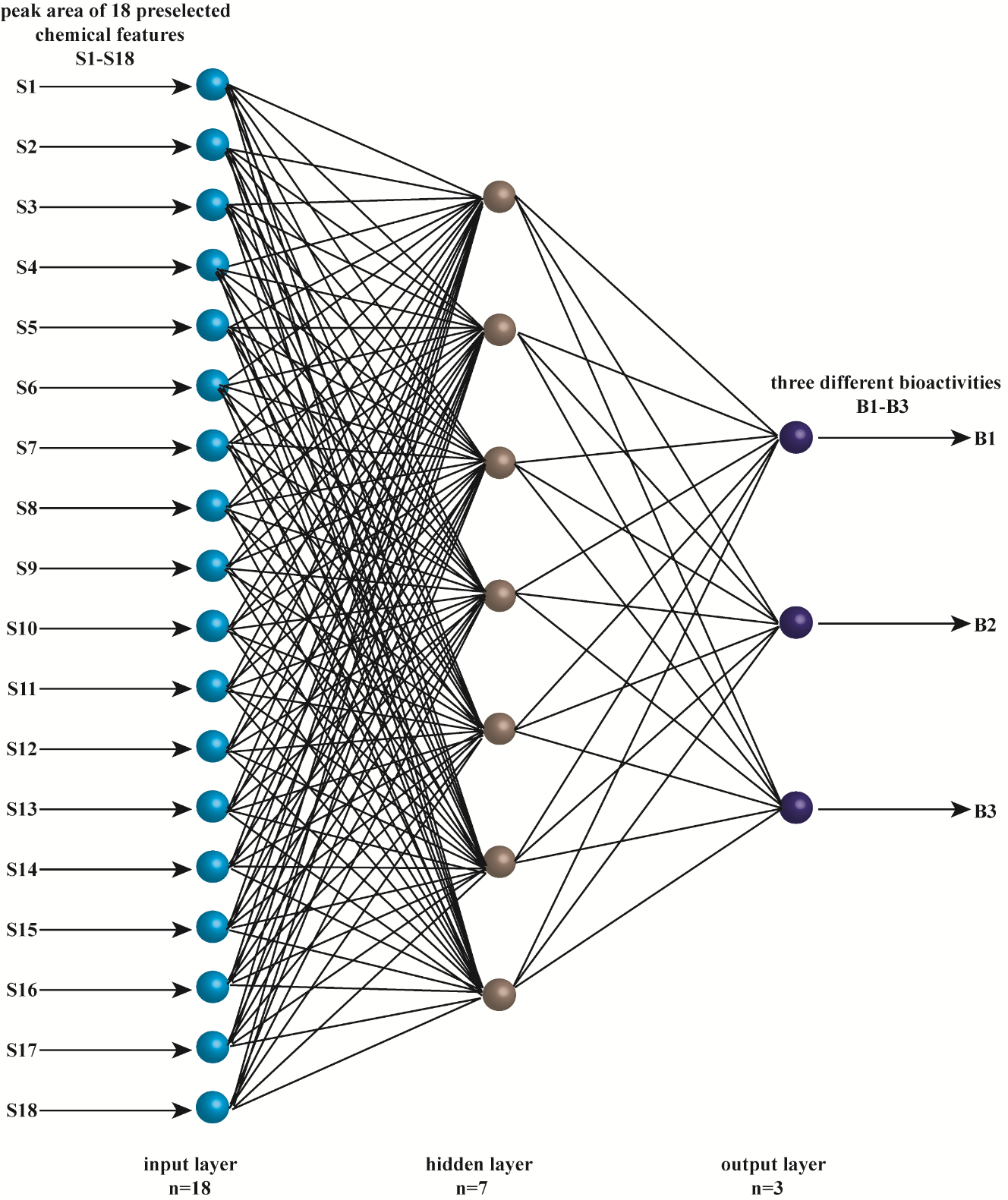


**Figure S1**. Architecture and configuration of a backpropagation-artificial neural network (BP-ANN) model.


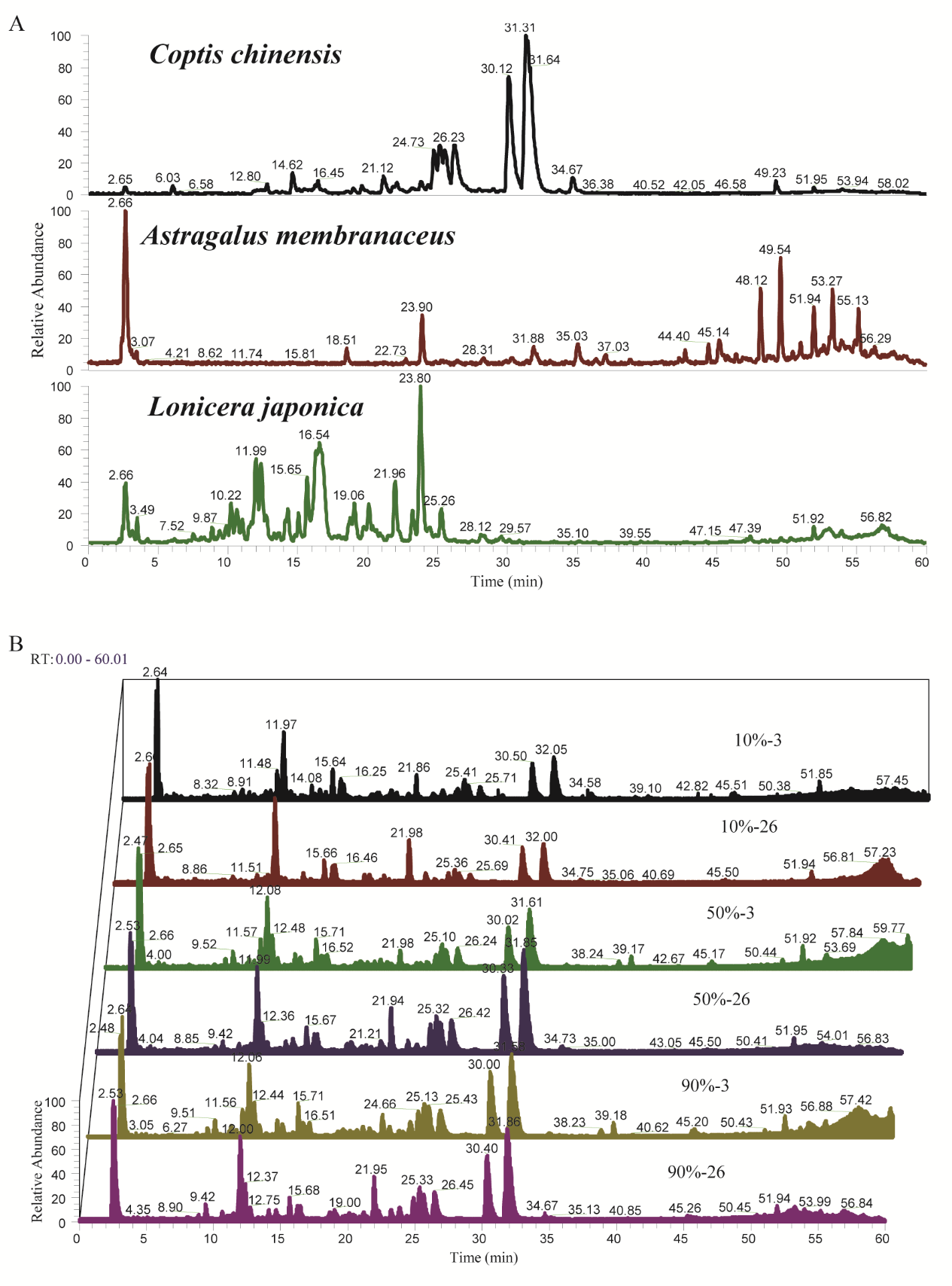


**Figure S2**. Representative total ion chromatograms obtained from different herbal medicines (A) and Jinqi Jiangtang (JQJT) preparations (B) based on UPLC-LTQ-Orbitrap.


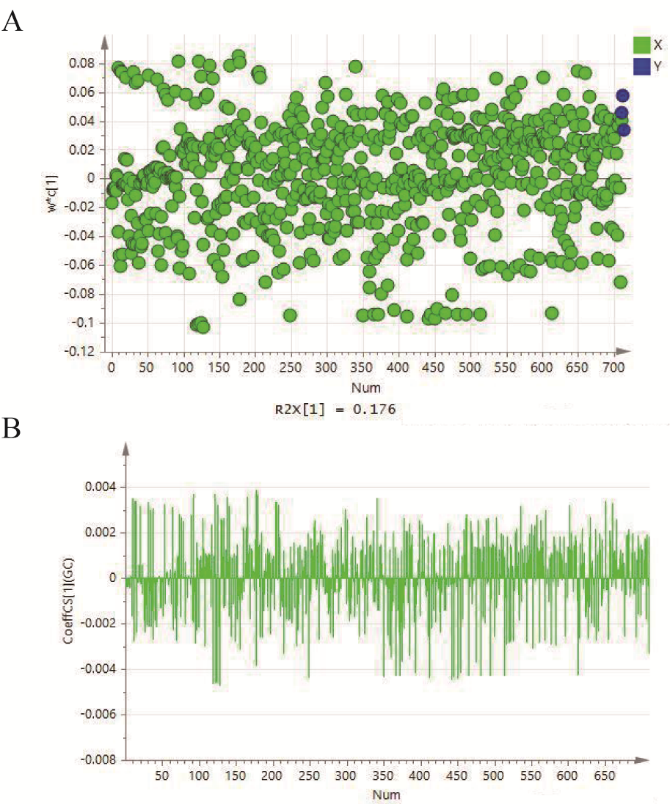


**Figure S3**. Regression coefficients obtained with partial least square (PLS) model.


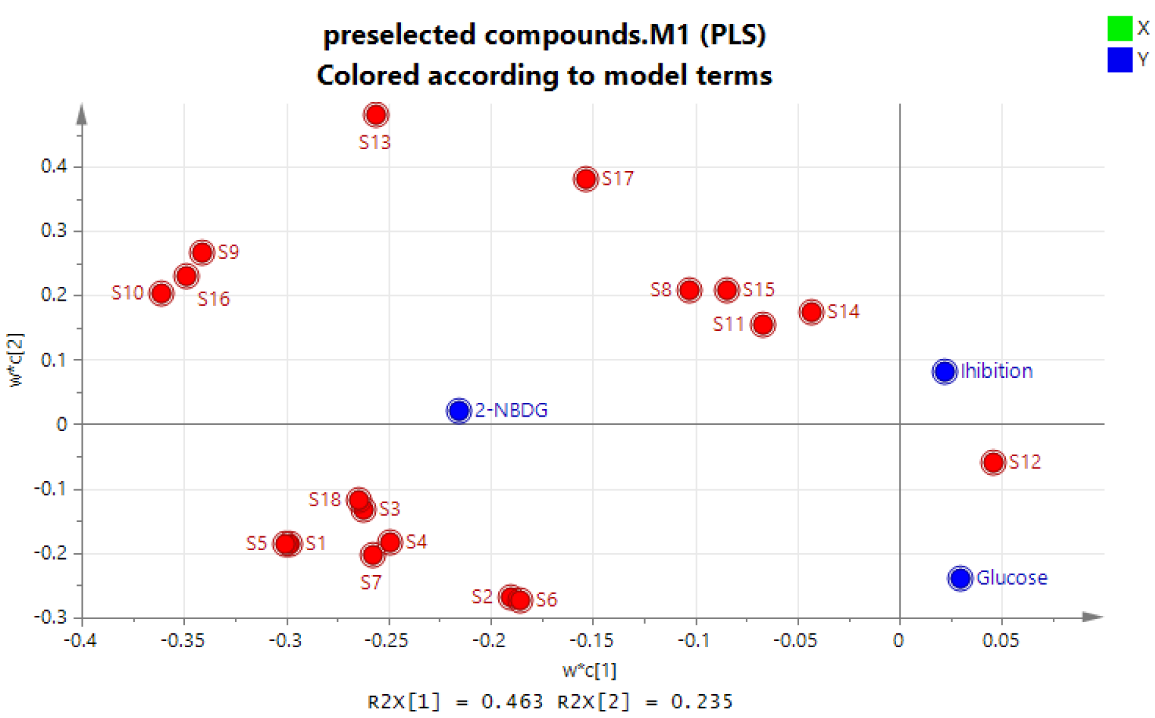


**Figure S4**. Regression coefficients obtained with PLS model.


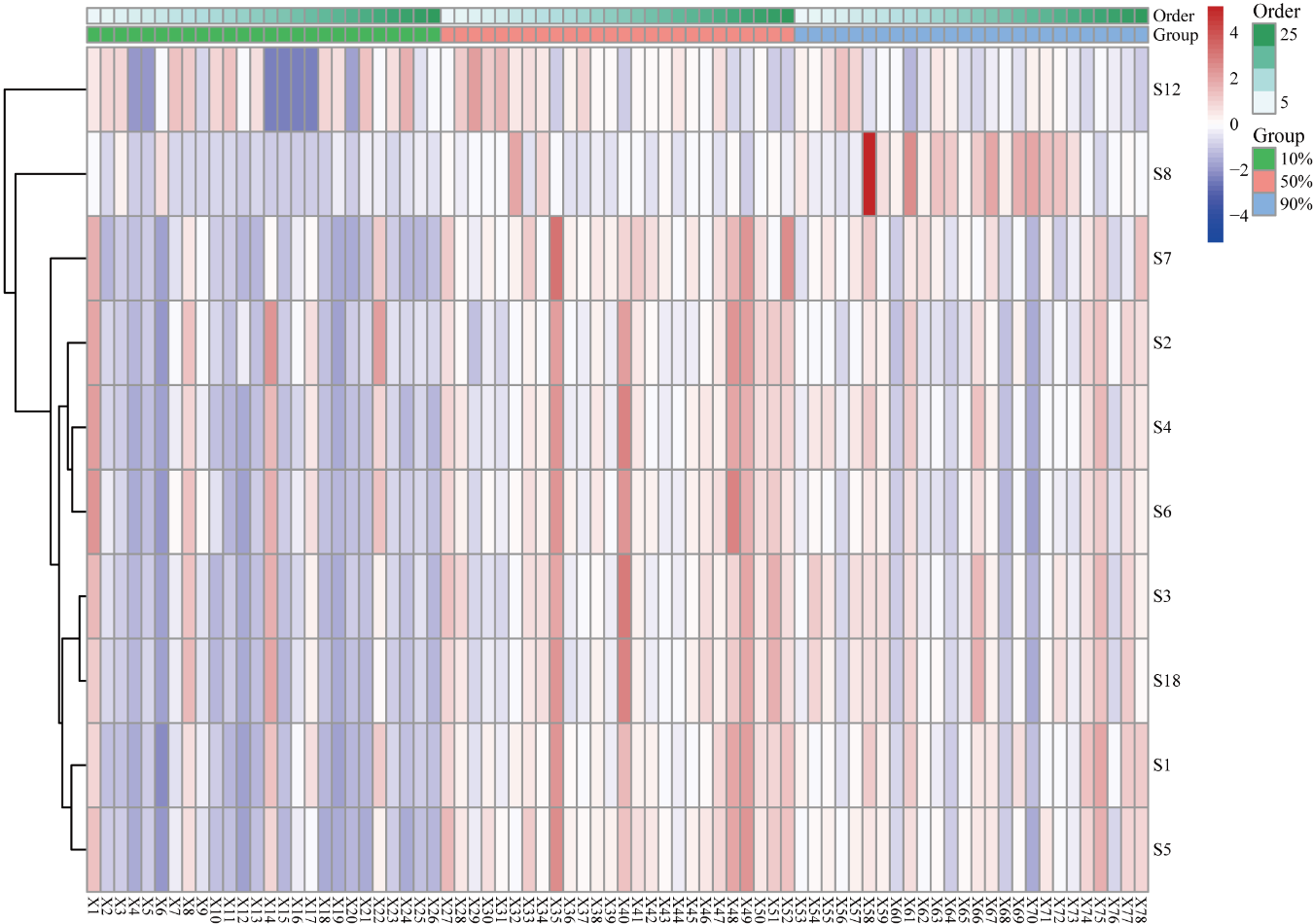


**Figure S5**. A heatmap based on content distribution of 10 screened Q-markers in 78 JQJT samples.

| **Backpropagation algorithm** | **Training function** | **Performance of BP-ANN model** | |
| --- | --- | --- | --- |
|  |  | **MSE** | **R** |
| BFGS quasi-Newton backpropagation | trainbfg | 0.19967 | 0.97358 |
| Bayesian regularization | trainbr | 0.0100288 | 0.99281 |
| Powell–Beale conjugate gradient backpropagation | traincgb | 0.19938 | 0.97949 |
| Fletcher–Reeves conjugate gradient backpropagation | traaincgf | 0.43224 | 0.96528 |
| Polak–Ribi’ere conjugate gradient backpropagation | traincgp | 0.26895 | 0.97545 |
| Batch gradient descent | traingd | 0.26932 | 0.96479 |
| Levenberg-Marquardt backpropagation | trainlm | 0.057722 | 0.99112 |
| Batch gradient descent with momentum | taningdm | 0.38351 | 0.97376 |
| Gradient descent with adaptive learning rate | traingda | 0.27686 | 0.97692 |
| Variable learning rate back propagation | traingdx | 0.26475 | 0.97265 |
| One step secant backpropagation | trainoss | 0.25393 | 0.97146 |
| Random Weight/Bias rule | trainr | 0.37336 | 0.96912 |
| RProp | trainrp | 0.57307 | 0.954 |
| Scaled conjugate gradient backpropagation | trainscg | 0.66747 | 0.95695 |

**Table S1.** Comparison of different backpropagation training algorithms.

**Table S2.** Comparison of transfer functions of BP-ANN model.

| **Transfer function** | **hidden layer (neuron numbers)** | **MSE** | **R (all)** |
| --- | --- | --- | --- |
| tansig | 7 | 0.0014 | 0.9983 |
| logsig | 7 | 0.0018 | 0.9978 |
| purelin | 7 | 0.001 | 0.9892 |
| tansig | 1 | 0.004 | 0.9952 |
| tansig | 5 | 0.0023 | 0.9975 |
| tansig | 10 | 0.0031 | 0.9951 |

**Table S3.** Functions and optimized parameters used in the establishment of BP-ANN model.

| **Training parameters of BP-ANN** | **Selected** |
| --- | --- |
| Transferfunction | Trainbr |
| network topologies | 18-7-3 |
| division function | *dividerand* |
| net.trainParam.epochs | 1000 |
| net.trainParam.goal | 0 |
| net.trainParam.max_fail | 10 |
| net.trainParam.min_grad | 1.00E-07 |
| net.trainParam.mu | 0.001 |
| net.trainParam.mu_dec | 0.1 |
| net.trainParam.mu_inc | 100 |
| net.trainParam.mu_max | 1.00E+10 |
| net.trainParam.show | 10 |
| net.trainParam.showCommandLine | FALSE |
| net.trainParam.showWindow | TRUE |
| net.trainParam.time | inf |
| initial bias | 0.1 |
| initial weights | 0.1 |

**Table S4.** The mean impact values (MIVs) of pre-selected chemical features obtained by BP-ANN model.

| **Pre-selected compounds** | **MIVs** | | | **Sum of coefficients** |
| --- | --- | --- | --- | --- |
|  | **Glucose consumption** | **Uptake of 2-NBDG** | **Inhibition** |  |
| S1 | 0.0014 | 0.0012 | 0 | 0.0026 |
| S2 | 0.0034 | 0 | 0 | 0.0034 |
| S3 | 0.00048 | 0.8577 | -0.0026 | 0.85558 |
| S4 | 0.0012 | 0.0005 | 0 | 0.0017 |
| S5 | 0.0009 | 0.0012 | 0 | 0.0021 |
| S6 | 0.0028 | -0.0001 | 0 | 0.0027 |
| S7 | 0.0014 | 0.0007 | 0 | 0.0021 |
| S8 | -0.0007 | 0.1556 | -0.0003 | 0.15457 |
| S9 | -0.0082 | 0.0038 | 0 | -0.0044 |
| S10 | -0.0045 | 0.0024 | 0 | -0.0021 |
| S11 | -0.0003 | -0.1534 | 0.0005 | -0.1532 |
| S12 | 0.0022 | -0.0008 | 0 | 0.0014 |
| S13 | -0.0036 | 0.0011 | 0 | -0.0025 |
| S14 | -0.0015 | 0.0003 | 0 | -0.0012 |
| S15 | -0.001 | 0.0002 | 0 | -0.0008 |
| S16 | -0.0056 | 0.0024 | 0 | -0.0032 |
| S17 | -0.0029 | 0.0005 | 0 | -0.0024 |
| S18 | 7.6E-05 | 0.9829 | -0.0029 | 0.98008 |
